# Application of *Bacillus amyloliquefaciens* CECT 5940 influenced muscle satellite cells, PCNA and acute phase protein secretion in broilers

**DOI:** 10.1007/s11259-025-10769-x

**Published:** 2025-05-21

**Authors:** Viera Karaffová, Renáta Szabóová, Csilla Tóthová, Rudolf Žitňan, Michaela Čechová, Martin Levkut, Erik Hudec, Zuzana Ševčíková, Monika Röntgen, Elke Albrecht, Róbert Herich

**Affiliations:** 1 Department of Morphological Disciplines, University of Veterinary Medicine and Pharmacy in Košice, Košice, Slovakia; 2 Department of Biology and Physiology, University of Veterinary Medicine in Košice, Košice, Slovak Republic; 3 Clinic of Ruminants, University of Veterinary Medicine and Pharmacy in Košice, Komenského 73, 041 81 Košice, Slovakia; 4 National Agriculture and Food Centre Research Institute of Animal Production, Nitra, Slovak Republic; 5 Research Institute for Farm Animal Biology (FBN), Dummerstorf, Germany

**Keywords:** *Bacillus amyloliquefaciens*, Growth factor, PCNA, Acute phase protein, Broiler

## Abstract

Despite the growing interest in the use of probiotics in broilers as feed additives, studies conducted to investigate the effect of probiotic administration on acute phase responses and the impact on muscle growth parameters in broilers are still limited. In this study, we investigated the effect of *Bacillus amyloliquefaciens* CECT 5940 on relative gene expression for growth factors involved in muscle development (insulin-like growth factors, myogenic factor 5, paired-box transcription factor), percentage of proliferating antigen cell nuclei in breast muscle, and secretion of acute phase proteins (serum amyloid A, haptoglobin, alpha_1_-acid glycoprotein) in the peripheral blood of broilers. Sixty one-day-old chicks from the experimental group were sprayed with a probiotic containing *B. amyloliquefaciens* at a dose of 1 × 10^10^ CFU/g directly after hatching and received the probiotic in drinking water (50 × 10^10^ CFU/1000 L) for 5 consecutive days of life. Sampling was performed on the 5th, 8th, and 12th day of life of the chicks. From the obtained results, we can conclude that *B. amyloliquefaciens* modulated the gene expression of selected growth parameters in the pectoral muscle, thereby increasing the number of satellite cells, however, their uptake into muscle fibers and thus increased hypertrophic growth was not proven at the time of the last sampling. At the same time, it demonstrated a potentiating effect on PCNA expression in chicken breast muscles in the early intensive growth phase of broiler chickens and modulated the production of acute phase proteins, which may contribute to improving adaptive processes during the growth and development of broilers.

## Introduction

Poultry meat is still a popular source of high-quality and easily digestible protein for most consumers today. For this reason, the poultry industry is under pressure to produce high-quality meat with minimal health risks for its consumers (Gaweł et al. 2022). In general, the meat quality of broiler is influenced by various factors, including nutrition, heredity, as well as environmental conditions. Among the most important factors is muscle development, which significantly determines meat properties such as texture, tenderness and nutritional composition (Yu et al. 2022).

At the molecular level, growth factors such as insulin-like growth factors (IGF-1 and IGF-2) and muscle regulatory factors like Myf-5 (myogenic factor 5) and PAX-7 (paired-box transcription factor) play a key role in regulating muscle growth, differentiation, and regeneration in poultry. IGF-1 and IGF-2 are essential components of the growth signaling system that promote protein synthesis and muscle cell proliferation. Moreover, in chickens, IGF-1 specifically regulates the increase in cellular protein content in myotubes and promotes myoblast proliferation (Fujita et al. 2019). The factors mentioned, including Myf-5 and PAX-7, are also crucial for satellite cell activity and muscle fiber development (Rahman et al. 2023; Ustanina et al. 2007).

Other important muscle markers include proliferating cell nuclear antigen (PCNA), the expression of which is increased in activated skeletal muscle satellite cells (Al-Zghoul and El-Bahr 2019). In poultry, PCNA expression can provide important information about muscle cell/satellite cell activity during growth, differentiation, and repair processes (Yin et al. 2013). Additionally, the number of satellite cells and PCNA expression in these cells of skeletal muscle tissue increase, especially during the rapid growth phase in broiler chickens and turkeys (Halevy 2020).

During this period, chickens are particularly susceptible to stress, which triggers an immune response and the production of acute phase proteins. This response also activates the hypothalamic-pituitary-adrenal axis, leading to various physical symptoms, including muscle cell catabolism (Gruys et al. 2005).

The acute phase response represents a crucial component of the innate immune system, acting as the first line of defence against infections, inflammation, trauma, and other stressors, with the goal of restoring homeostasis (Cray et al. 2009). It involves systemic biochemical and metabolic changes, including altered production of liver-derived acute phase proteins. In poultry, the most prominent acute phase proteins are α1-acid glycoprotein (AGP), serum amyloid A (SAA), ceruloplasmin, transferrin, haptoglobin (Hp), fibrinogen, and fibronectin (O’Reilly et al. 2018). However, excessive or prolonged activation of these proteins may lead to adverse effects, such as anorexia, fever, weakness, amyloidosis, and muscle wasting (cachexia) (Gulhar et al. 2023). Thus, strategies to modulate the acute phase response, including dietary supplementation with probiotics, could help maintain immune balance and overall homeostasis in poultry.

Probiotic bacteria have recently been used to support the overall health of broiler chickens, including improving the quality of the meat produced. Recent studies indicated that supplementation of the probiotic strain *Bacillus amyloliquefaciens* CECT 5940 in the diet of broilers can improve growth performance, as it had a positive effect on intestinal health, nutrient absorption, immunity and overall animal performance (Sun et al. 2022). Moreover, recent studies pointed out the fact that various probiotic strains might have affected the expression of genes involved in muscle development, which consequently influenced the final quality of meat (Muyyarikkandy et al. 2023; Albrecht et al. 2022; Stasiak et al. 2021).

Although interest in using probiotics as feed additives for broiler chickens is increasing, there are still only a few studies examining their effects on acute phase responses and muscle growth factors. Therefore, in this study, we decided to observe the effect of *Bacillus amyloliquefaciens* CECT 5940 on the relative gene expression for growth factors involved in muscle development (IGF-1, IGF-2, Myf-5, PAX-7), the percentage of PCNA positive nuclei in the *pectoralis major* muscle, and the secretion of acute phase proteins (SAA, Hp, AGP) in the peripheral blood of broilers.

## Materials and methods

### Animals

Broiler chickens were handled and killed using procedures in accordance with state regulations. The experiment was approved by the Ethical Committee of Veterinary Medicine and Pharmacy in Košice and the Animal Welfare Commission of the Ministry of Agriculture of the Slovak Republic (EKVP/2023-18). Birds had free access to feed commercial BR1 starter feed mixtures for broiler (Table 1) and water. In addition, all recommended welfare requirements for broiler chickens were met.

**Table 1:** Composition of BR1 commercial diet for broilers

Ingredients g/kg	BR1
Wheat	290
Maize	300
Soybean meal	320
Rapeseed oil	40
Fish meal	20
Limestone	12
Dicalcium phosphate	10
Sodium chloride	2
DL-methionine	1
Vitamin-mineral mix^*^	5
Composition by analysis (g/kg)	
dry matter	899.9
crude protein	232.7
fat	64.5
dietary fibre	22.7
ash	53
Ca (calcium)	90.4
P (phosphorus) total	69.6

Vitamin and mineral premix^*^: vitamin A 12,500 IU/kg, vitamin D3 4,000 IU/kg, vitamin E 80.00 mg/kg, Cu 15.00 mg/kg, vitamin D/25 cholekalciferol 1,000 IU/kg, Jod 1.00 mg/kg, Mn 50.00 mg/kg, Zn 90.00 mg/kg, Fe 40.00 mg/kg, Se 30.00 mg/kg

### Experimental scheme

The experiment was performed in a broiler farm using two barns for 20,000 chickens of the broiler breed ROSS 308 each. Sixty one-day-old chickens of the experimental (E) group were sprayed with the probiotic ECOBIOL Soluble plus (Evonik) *Bacillus amyloliquefaciens* CECT 5940 (1 × 10^10^ CFU/g) directly after hatch and received the probiotic during the consecutive 5 days of life via drinking water (50 × 10^10^ CFU/1000 L). The animals from the control (C) group received 10 mg enrofloxacin/kg life weight (ENROGAL, Pharmagal, Slovakia, 100 mg/mL) via drinking water during the first four days of life. All animals were supplied with additional vitamins and minerals (Vitaplan DCP, vitamin D3, Ca, P) at day 5 to 7 of life via drinking water (350 mL/1000 L) over 12 h. At day 10 to 12 of life, all animals received Hepavet via drinking water (500 mL/1000 L) over 12 h.

Each ten chickens (*n* = 10) per group were randomly selected at day 5, 8, and 12 of life and slaughtered in an approved manner. Body weight was recorded on each day of collection. The *pectoralis major* muscle was weighed on the last day of collection and samples were taken for further analysis. Peripheral blood was collected in a similar manner for further analyses as described below.

### Sample homogenization and isolation of total RNA for selected genes

The *pectoralis major* muscle samples weighing 20 mg pieces were immediately placed in RNA Later solution (Qiagen, Hilden, Germany) and stored at −70 °C. RNA purification and transcription were then performed as described in Karaffová et al. 2019.

### Quantitative real time-qPCR

The mRNA levels of IGF-1, IGF-2, Myf-5 and PAX-7 genes in breast muscle of broiler chickens (*n = 10*) were determined and normalized to the reference gene GAPDH (glyceraldehyde-3-phosphate dehydrogenase). GAPDH was selected based on confirmed expression stability using geNorm software (Vandesompele et al. 2002).

Amplification and detection of target products were performed using SsoAdvanced Universal SYBR Green Supermix (Bio-Rad, Hercules, California) and specific primers (Table 2) on the LightCycler 480 II instrument (Roche, Basel, Switzerland). Each qReal-Time PCR for relative level detection was run under the following conditions: initial denaturation at 95 °C for 5 min followed by 38 cycles: denaturation at 95 °C for 15 s, annealing (Table 2) and elongation for 2 min at 72 °C. A melting curve from 55 °C to 95 °C with readings at every 0.5 °C was recorded for each individual qReal-Time PCR plate. All reactions for qReal-Time PCR were performed in duplicate and individual primer sets allowed for cDNA amplification efficiencies between 94% and 100%. The amplification efficiency for each target gene (including GAPDH) was confirmed to be essentially 100% in the exponential phase of the reaction, where the quantification cycle (Cq) was calculated. The Cq values of the studied genes were normalised to an average Cq value of the reference gene (^Δ^Cq), and the relative expression of each gene was calculated mathematically as 2–^ΔCq^.

**Table 2:** List of primers used for the gene quantification in *pectoralis major* muscle of broiler chickens

Primer	Sequence 5’–3’	Annealing temperature/time	References
IGF-1 Fw	GAGCTGGTTGATGCTCTTCAGTT	60 °C/30 s	Xiao et al. 2017
IGF-1 Rev	CCAGCCTCCTCAGGTCACAACT		
IGF-2 Fw	CTCTGCTGGAAACCTACTGT	54 °C/30 s	Mudroňová et al. 2018
IGF-2 Rev	GAGTACTTGGCATGAGATGG		
Myf-5 Fw	CAGAGACTCCCCAAAGTGGAGAT	60 °C/30 s	Xiao et al. 2017
Myf-5 Rev	GTCCCGGCAGGTGATAGTAGTTC		
PAX-7 Fw	AGGCTGACTTCTCCATCTCTCCT		Adhikari et al. 2020
PAX-7 Rev	TGTAACTGGTGGTGCTGTAGGTG		
GAPDH Fw	CCTGCATCTGCCCATTT	59 °C/30 s	De Boever et al. 2008
GAPDH Rev	GGCACGCCATCACTATC		

### Histomorphometric detection and quantification of PCNA in breast muscle

Chicken breast muscle samples (*n = 10*) measuring 1 cm^3^ were obtained from the *musculus pectoralis major* region and immediately fixed in 4% formaldehyde, dehydrated, and embedded in paraffin. The paraffin blocks – muscle tissue sections were cut at 5 μm (sledge microtome, Leica, Germany), placed on glass slides, deparaffinised and rehydrated as previously described in Koivukoski et al. 2023. Sections were immunostained with primary monoclonal antibody used for chicken species such as proliferating cell nuclear antigen (PCNA antibody; SPM350), a molecular marker - protein for determination of proliferation cell activity, using a commercial kits (Novus Biologicals, Toronto, Canada) and secondary anti-mouse antibody such as biotinylation reagent (Dako, Glostrup, Denmark) according to the manufacturer’s protocol with final staining of tissue slides in hematoxylin bath. The negative control was obtained by the same methodology, but without using the primary PCNA antibody on tissue sections.

Histomorphometric analysis was obtained as number of the PCNA-positive nuclei (in %) of white muscle (*m. pectoralis major*) in seven random fields of view (400 x magnification, Nikon Labophot 2 microscope, Nikon Corporation, Japan) of each sample of tissue sections from the E and/or C group using Nikon DS-Fi1 camera (Nikon Corporation, Japan) with software NIS-Elements Advanced Research (version 3.0, Laboratory Imaging, Czech Republic). The percentage of PCNA-expressing nuclei for each tissue section sample was determined as the number of PCNA-expressing nuclei divided by the total number of cells multiplied by 100 (Guo et al. 2011).

### Laboratory analyses of acute phase proteins

The blood samples (*n = 10*) for the analysis of acute phase proteins were taken into 1.1 mL serum gel separator tubes without additives and anticoagulants (Sarstedt, Nümbrecht, Germany). After letting the blood samples to coagulate at room temperature, sera were separated by centrifugation at 3,000 × g for 15 min and then transferred into Eppendorf tubes. The serum samples were immediately processed and analyzed, and aliquots were kept frozen at −20 °C for further laboratory analyses.

The serum samples were analyzed for the concentrations of selected inflammatory proteins: serum amyloid A (SAA, ng/ml), alpha_1_-acid glycoprotein (AGP, mg/mL), and haptoglobin (Hp, mg/mL). The concentrations of SAA were quantified by double antibody sandwich enzyme linked immunosorbent assay (ELISA) using commercially available Chicken SAA ELISA kit (Immunology Consultants Laboratory, Inc., Portland, OR, USA). The values of AGP were determined by commercial Chicken AGP ELISA kit AGP-5 (Life Diagnostics, Inc., West Chester, PA, USA). Haptoglobin (Hp, mg/ml) was measured spectrophotometrically using commercial colorimetric kits (Tridelta Development, Kildare, Ireland) in microplates. The absorbance was read on Opsys MR automatic microplate reader (The Dynex Technologies, USA) at a wavelength determined by the manufacturers of the kits. The Revelation QuickLink version 4.25 computer software was used for the calculation of results (The Dynex Technologies, USA).

## Statistical analyses

### Histomorphometric detection and quantification of PCNA and gene expression in breast muscle

Statistical analysis was performed using the Student’s paired t-test using Graph Pad Prism version 8.00 (GraphPad Software Inc., California, USA) to determine differences between each group. Differences between the mean values for each group were considered statistically significant at *P* < 0.05^*^; *P* < 0.01^**^; *P* < 0.001^***^; *P* < 0.0001^****^. Values in figures are given as means with standard deviations (± SD).

### Laboratory analyses of acute phase proteins

Mean values and standard deviations were calculated using descriptive statistics in the computer program GraphPad Prism V5.02 (GraphPad Software Inc., California, USA). Kolmogorov-Smirnov test for normality was used to evaluate the distribution of the data. The overall variances throughout the evaluated period in both groups of chickens were estimated using One-Way Analysis of Variance ANOVA Test. Unpaired t-test was applied to test the significance of differences between the C and E group for each evaluated protein. Differences were considered significant when the *p* value was less than *0.05*.

## Results

### Weight measurement

A gradual increase in total body weight was recorded in both experimental groups. However, on the other hand, in the experimental group of broiler chickens, there was a significant increase in body weight (*P < 0.001*) on the 12 th day of age compared to the C group (Table 3). Weight of the *pectoralis major* muscle was not different in the experimental group on the last day of collection compared to the C group (Table 3).

**Table 3:** Body weight and *pectoralis major* muscle weight of C group and *B*. *amyloliquefaciens* CECT 5940 treated broiler chicken during sample collection

Weight	Group	Day of age		
		5 day	8 day	12 day
Body	C	89.3 ± 5	169.8 ± 7.7	318 ± 13.4
	E	95.5 ± 6.3	161.5 ± 4.9	347.9 ± 9.3^***^
*Musculus * *pectoralis * *major*	C	7.4 ± 0.8	23.5 ± 2.3	61.1 ± 3.5
	E	7.7 ± 0.9	23.6 ± 2.2	62.3 ± 2.7

Differences between the mean values for each group were considered statistically significant at *P* < 0.001^***^. Values in figures are given as means with standard deviations (± SD)

### Quantitative real time-qPCR

**Fig. 1:**
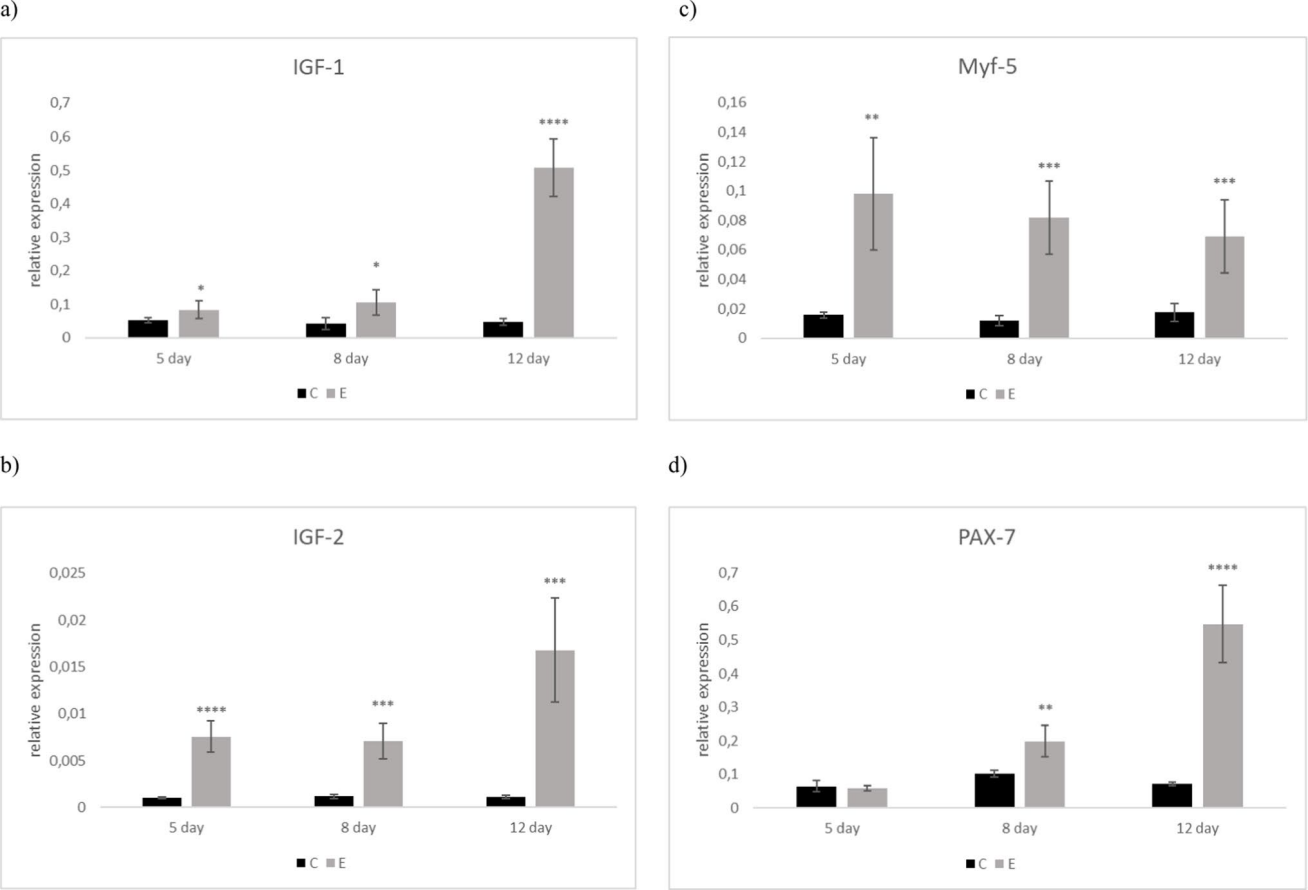
Relative gene expression for selected parameters in *pectoralis major* muscle of broiler chicken: (**a**) IGF-1, (**b**) IGF-2, (**c**) Myf-5, (**d**) PAX-7. ^*^ different asterisk indicate significant differences among groups and time points at *P* < 0.05; ^**^ different asterisk indicate significant differences among groups and time points at *P* < 0.01; ^***^different asterisk indicate significant differences among groups and time points at P < 0.001;^****^different asterisk indicate significant differences among groups and time points at *P < 0.0001*

The relative IGF-1 gene expression was markedly upregulated in experimental group in comparison with C group during all collection days (*P < 0.05; P < 0.0001*) (Fig. 1a). The same tendency was observed for IGF-2 gene expression, which was the highest on the 12 th day of age in E group compared to the C group (*P < 0.001*) (Fig. 1b). Similarly, Myf-5 gene expression was significantly upregulated in E group in comparison with control at different levels of significance (*P < 0.01; P < 0.001*) during all collections (Fig. 1c). PAX-7 relative gene expression was upregulated in E group at 7 day of age (*P < 0.01*) as well as on the 12 th day of age (*P < 0.0001*) compared to the C group (Fig. 1d).

### Histomorphometric detection and quantification of PCNA in breast muscle

**Table 4:** Differences in the percentage of PCNA-expressing nuclei in the E and C group of broiler chickens (mean ± sd)

Day of age	C	E
Day 5	82.30 ± 11.09	**93.26 ± 1.64** ^*^
Day 8	62.61 ± 11.93	69.97 ± 4.62
Day 12	54.74 ± 9.19	51.29 ± 5.79

Differences between the mean values for each group were considered statistically significant at *P* < 0.05^*^

After 4-day ECOBIOL Soluble Plus administration period (5 day), the significant PCNA expression (*P < 0.05*) in chicken muscle cells was observed in experimental group compared to the C group. In like manner, we observed increased percentage of PCNA-positive nuclei in E group with comparison to the C group on the 8 th day of age (Table 4).

### Laboratory analyses of acute phase proteins

The concentrations of SAA were significantly lower in the experimental group for each sample collection days (*P < 0.01* and *P < 0.001*, respectively). For the concentrations of Hp, significant differences were obtained only on the 12 th day of age, with lower values in the experimental group (*P < 0.05*). An opposite trend was observed in the concentrations of AGP, showing significantly higher mean value in the experimental group on the 12 th day of age (*P < 0.001*).

The analysis of the concentrations of acute phase proteins throughout the evaluated period showed in the experimental group of chickens no significant differences for the concentrations of SAA and Hp. Significant differences in the experimental chickens were found in the concentrations of AGP (*P < 0.05*), with a trend of increasing values with age.

In the C group of chickens, significant variations throughout the evaluated period were observed in the concentrations of SAA (*P < 0.001*), with the highest mean value on day 5 of age. An opposite trend of significantly increasing values was observed for Hp (*P < 0.05*), while the concentrations of AGP showed no significant variations throughout the evaluated period of life (Table 5).

**Table 5:** Differences in the concentrations of evaluated acute phase proteins between the E and C group of broiler chickens, and during the evaluated period (mean ± sd)

Parameter		Day of age			*P* value ANOVA
		Day 5	Day 8	Day 12	
SAA (ng/ml)	C	53.65 ± 15.95^a^	35.83 ± 7.12^a^	34.54 ± 6.81^c^	< 0.001
	E	26.35 ± 3.46^b^	24.31 ± 3.39^b^	25.86 ± 4.28^d^	n.s.
Hp (mg/ml)	C	0.053 ± 0.3	0.099 ± 0.07	0.096 ± 0.04^a^	< 0.05
	E	0.046 ± 0.4	0.054 ± 0.05	0.049 ± 0.04^b^	n.s.
AGP (mg/ml)	C	0.229 ± 0.07	0.281 ± 0.10	0.211 ± 0.05^e^	n.s.
	E	0.247 ± 0.10	0.360 ± 0.08	0.362 ± 0.09^f^	< 0.05

*P* value - significance of the ANOVA test (*P < 0.05* and *P* < 0.001); x – mean value; sd - standard deviation; n.s. –not significant; ^a, b,c, d,e, f^ - different superscripts in columns mean statistically significant difference between the groups (^a, b^*P < 0.001*; ^c, d^*P* < 0.01; ^e, f^*P* < 0.05)

## Discussion

Several studies have shown that changes in gut microbiota composition can influence nutrient metabolism, leading to increased muscle mass and altered meat quality in livestock (Park and Kim 2014; Abdulla et al. 2017; Žitňan et al. 2019). Probiotics can modulate the host’s gut microbiota, limit pathogenic bacteria, and improve nutrient absorption, which may enhance animal growth (Younis et al. 2013; Olnood et al. 2015; Maiorano et al. 2017). However, their effects on muscle growth remain inconsistent (Niewold 2007; Albrecht et al. 2022).

As we know, in mammals IGF-1 contributes to growth and body weight gain, while its insufficient production is associated with dwarfism (Yakar et al. 2002). In chickens, however, the application of synthetic IGF-1 caused an increase in the cellular protein content in myotubes, confirming the direct myogenic effect of the growth factor (Nakashima et al. 2017). Along with previous, a proliferative effect on chicken myoblasts was observed (Yu et al. 2015). On the other hand, a direct correlation between IGF and growth in birds has not been clearly described so far (Vaccaro et al. 2024).

Our results showed a gradual increase in gene expression for almost all investigated growth factors, except for Myf-5 in the experimental group, which may have indicated a cumulative effect of the applied probiotic strain. This could also have been reflected in the increased total weight of the chickens on the last day of collection (day 12 of age), although the weight of the pectoral muscle was not higher compared to the control group. This may have suggested that a longer duration of probiotic administration was required to achieve a significant effect of *B*. *amyloliquefaciens* CECT 5940 on pectoral muscle mass, as the standard length of fattening is around 42 days (Dağdemir et al. 2007). In contrast, the application of the probiotic preparation caused an increase in the expression of the Myf-5 gene compared to the C group, but in the experimental group, it showed an overall decreasing trend with increasing age of the chickens. Our results were consistent with studies confirming that the level of Myf-5 gene expression decreased with age (Ancel et al. 2024; Yamamoto et al. 2018).

On the other hand, the increase in IGF-1 and PAX-7 expression in the pectoral muscle at the last sampling in the experimental group may reflect a reduced differentiation potential of myogenesis.

Myf-5 and PAX-7 are key regulators of embryonic myogenesis, but they also remain important after hatching, when muscle fibers continue to grow by increasing protein content and nuclear number (Halevy 2020). A deficiency in PAX-7 leads to early depletion of satellite cells during postnatal growth (Day et al. 2009). Similarly, our previous study (Žitňan et al., 2019) highlighted the early post-hatch period as critical for satellite cell activity and muscle development.

Based on this, we hypothesize that *B. amyloliquefaciens* may influence the expression of key genes regulating satellite cell activity (PAX-7, Myf-5) and postnatal muscle growth (IGF-1, IGF-2). In contrast to our previous findings with *E. faecium* AL41, where IGF-1 expression declined with age (Albrecht et al. 2022), the current study showed sustained IGF expression in the probiotic group. This may reflect strain-specific mechanisms. The absence of a late-stage IGF-1 decline, which is needed for satellite cell fusion (Yoshida and Delafontaine 2020), suggests no clear stimulation of muscle fiber growth.

Our hypothesis is supported by a study in which the administration of another probiotic preparation, containing mainly the genera *Lactobacillus*,* Lactococcus*, and *Bifidobacterium*, did not improve the quality or growth of the pectoralis major muscle in 42-day-old broiler chickens (Stęczny and Kokoszynski 2019).

Taken into account that the probiotic effect depends on the dose, application method, strain interactions, and duration of use, it is important to note that only a limited number of studies have investigated their influence on the expression of myogenic growth factors, which may be one of the key mechanisms affecting muscle development in meat-producing poultry.

PCNA is a well-established marker of active cell proliferation and is highly expressed in satellite cells, particularly in broiler chickens selected for rapid growth and high muscle yield (Zeng et al. 2020; Ferreira et al. 2020). In our study, dietary supplementation with the probiotic strain *Bacillus amyloliquefaciens* CECT 5940 led to an increased expression of PCNA in the breast muscle of broilers at 5 days of age, a key period of early intensive growth. This suggests a higher presence or activation of satellite cells at this stage.

However, the elevated PCNA expression did not result in a corresponding increase in muscle fiber size or breast muscle mass, indicating that the additional satellite cells may not have been effectively incorporated into muscle fibers. Similar findings were reported in a study by Chen et al. (2016), where supplementation with *Lactobacillus plantarum* TWK10 in mice enhanced muscle mass and strength, suggesting that probiotic effects on muscle development may vary by strain and species.

Overall, our results suggest that while the tested probiotic strain may support satellite cell activity, it does not act as a direct stimulator of hypertrophic muscle growth in broiler chickens under the given experimental conditions.

In our study, we observed lower values ​​of SAA and Hp on each sampling day in the experimental group. However, AGP levels were overall higher compared to the C group, which may indicate the modulatory activity of *B. amyloliquefaciens* CECT 5940, potentially contributing to improved adaptive processes during the growth and development of chickens. This assumption is further supported by the fact that human AGP is considered a natural anti-inflammatory protein due to its ability to modulate neutrophil chemotactic migration and superoxide generation in a concentration-dependent manner, thereby helping to maintain homeostasis (Khalil and Al-Humadi 2020).

The concentrations of selected acute phase proteins in the C group fluctuated during the monitored period, likely reflecting physiological metabolic changes linked to intensive growth rather than pathological processes. Similar age-related changes were observed by O’Reilly et al. (2018), particularly elevated SAA and ceruloplasmin in younger broilers. In contrast, studies by Kefal and Toker (2006) and Karaffová et al. (2022) reported no significant effects of probiotic supplementation on inflammatory markers, possibly due to differences in probiotic strain composition and mechanisms of action. However, further studies are necessary to achieve detailed results on the mechanism of influence of individual probiotic strains.

## Conclusion

In summary, our study demonstrates that *Bacillus amyloliquefaciens* CECT 5940 modulates the expression of genes associated with muscle growth in broiler chickens, increases the number of satellite cells, and enhances PCNA expression during the early intensive growth phase, although a clear effect on muscle fiber hypertrophy was not observed by the last sampling. These findings advance the understanding of how commercial probiotic preparations may influence muscle development and meat production in poultry, providing valuable insights that could be of particular interest to poultry breeders and producers.

## Data Availability

No datasets were generated or analysed during the current study.
